# Characteristics and Trends of Pneumoconiosis in the Jiangsu Province, China, 2006–2017

**DOI:** 10.3390/ijerph16030437

**Published:** 2019-02-02

**Authors:** Lei Han, Wenxi Yao, Zilong Bian, Yuan Zhao, Hengdong Zhang, Bangmei Ding, Han Shen, Ping Li, Baoli Zhu, Chunhui Ni

**Affiliations:** 1 Institute of Occupational Disease Prevention, Jiangsu Provincial Center for Disease Control and Prevention, Nanjing 210028, China; hanlei@jscdc.cn (L.H.); Kathy21143@hotmail.com (Y.Z.); Hd-zhang@263.net (H.Z.); dingbangmei@163.com (B.D.); shenhan411@163.com (H.S.); 2 Center for Global Health, School of Public Health, Nanjing Medical University, Nanjing 211166, China; ywx3737@163.com (W.Y.); ZilongBian@126.com (Z.B.); NJMUliping@163.com (P.L.); 3 Wuxi School of Medicine, Jiangnan University, Wuxi 214122, China

**Keywords:** occupational disease, pneumoconiosis, epidemiology

## Abstract

This study aims to describe the characteristics and trends of pneumoconiosis in the Jiangsu Province, China, and provide information for the occupational diseases control. We collected and analyzed the data of pneumoconiosis cases reported annually from 2006 to 2017. The information of the cases mainly includes case distributions, clinical types and stages, enterprise types and scales, as well as diagnosis age and exposure duration. A total of 9243 pneumoconiosis cases were reported between 2006 and 2017, among which silicosis and coal workers’ pneumoconiosis accounted for the vast majority (87.5%). The incidence of pneumoconiosis was relatively higher in Wuxi, Yancheng, Suzhou and Xuzhou, compared to the other district. Most pneumoconiosis cases occurred in the state-owned (58.4%) and collective enterprises (23.8%). Most cases worked in industries related to geology and coal production. The median exposure duration and diagnosis age of the total pneumoconiosis cases was 13.2 and 61.0 years, respectively. Therefore, more measurements are needed to control pneumoconiosis in the Jiangsu Province.

## 1. Introduction

Pneumoconiosis is a group of serious occupational diseases associated with the inhalation of mineral dusts and the corresponding reaction of lung tissue, which eventually induce irreversible lung damage [1]. The most common types of pneumoconiosis include silicosis, coal workers’ pneumoconiosis (CWP) and asbestosis [2]. Each year, approximately 125,000 cases of global deaths resulted from pneumoconiosis according to the Global Burden of Disease (GBD) study 2010 [3]. In America, pneumoconiosis caused 1000 to 2000 hospitalizations per year and accounted for 525 deaths in 2007 [4]. In Korea, there were 17,546 pneumoconiosis patients and about 3900 at-risk patients in 2008 [5]. However, the prevalence of pneumoconiosis in developing countries was even more severe. Between 1975 and 2007 in South Africa, the proportions of white and black gold mine workers with silicosis increased from 18 to 22% and from 3 to 32%, respectively [6]. Since the discovery of pneumoconiosis in the 19th century, the prevention of occupational diseases mainly concentrated on the control of dust-caused occupational hazards. The Joint ILO/WHO Committee on Occupational Health established the ILO/WHO Global Program for the Elimination of Silicosis (GPES) following the recommendation of the 12th Session in 1995, which aimed to call on the world to take steps for silicosis prevention [7]. A large number of researches and work had been done in dust control. To a certain extent, pneumoconiosis has been improved with the efforts of many countries. However, it remains a serious public health problem, especially in some developing countries [3,5,8,9,10,11].

Although multiple measurements have been applied to prevent and control pneumoconiosis during the past few decades in China, the incidence of pneumoconiosis was still at a high level. WHO reports showed that more than 500,000 silicosis patients were recorded in China between 1991 and 1995, and 6000 new cases and over 24,000 deaths had been recorded each year [12]. From 1997 to 2009, a total of 122,333 new pneumoconiosis cases were reported, 87.5% of which were attributed to the CWP and silicosis. The prevalence of pneumoconiosis showed an increasing tendency among workers exposed to occupational dust [13]. The National Occupational Disease Report of 2016 indicated that 26,873 new cases of pneumoconiosis were recorded in China, of which 54.3% were CWP and 39.7% were silicosis. The Jiangsu Province Occupational Disease Report in 2015 showed that new pneumoconiosis cases accounted for 65.32% of the total new occupational disease cases. Silicosis is the most common type of pneumoconiosis in the Jiangsu Province and accounted for more than 70% of total pneumoconiosis cases [14]. However, CWP cases were more commonly seen in Hunan, Chongqing, Shanxi and other provinces, which was due to the differences in the coal mines distribution and the composition of industry [15].

Since pneumoconiosis displayed different characteristics in different provinces of China, targeted analysis of the existing circumstance in Jiangsu Province is beneficial for the prevention and control of pneumoconiosis. Here, we analyzed the pneumoconiosis data of reported in the Jiangsu Province from 2006 to 2017 (12 years), and then demonstrated the characteristics and trends of pneumoconiosis, as well as the occupations and population of pneumoconiosis patients. Our study not only provided valuable information for the control and prevention of pneumoconiosis, but also facilitated the establishment of the time series model of pneumoconiosis monitoring and early warning. 

## 2. Materials and Methods

### 2.1. Sources of Data 

Since 2006, in order to manage occupational disease report data by networks and computers, the information of confirmed occupational diseases have been submitted by diagnosis institutions through Network Direct Report System of Occupational Diseases according to the requirements of the Ministry of Health. We acquired data of pneumoconiosis cases from 1 January 2006 to 31 December 2017 via the Health Hazards Monitoring System which is part of the National Disease Control and Prevention Information System.

Pneumoconiosis patients were identified through routine surveillance and occupational health examination each year. In China, the diagnoses for pneumoconiosis were performed by at least three radiologists according to the Diagnostic Criteria of Pneumoconiosis. Since the criteria were revised in 2002 and 2009, cases reported before 1 November 2009 were diagnosed according to the GBZ70-2002 version, and other cases were based on the GBZ70-2009 version. Pneumoconiosis was clearly divided into three stages in the GBZ70-2009 version. At the same time, the term “observation objects” was added to GBZ70-2009, which is defined as the discovery of uncertain pneumoconiosis-like changes on X-ray chest radiographs of dust workers, and the nature and extent of these changes require dynamic observation within a certain period of time.

### 2.2. Definition

Ownership type: Enterprises are divided into 6 categories based on ownership of the major paid-in capital: state-owned, collectively owned, privately owned, foreign, Hong Kong/Macao/Taiwan and others.

Enterprise scale: Based on the Enterprise Scale Standard established by the State Statistical Bureau, enterprises are divided into large, medium, small and micro (Table S1). As shown in Table S1, medium-sized and small businesses were required to meet all the conditions of operation revenue, employees and assets, while large and micro businesses could meet one of them.

In China, pneumoconiosis is classified into 13 categories, including silicosis, CWP, welders’ pneumoconiosis and cement pneumoconiosis, etc. According to the National Occupational Disease Classification and Catalogue, and the Diagnostic Criteria of Pneumoconiosis, welders’ pneumoconiosis is defined as a chronic pulmonary fibrosis disease caused by the long-term inhalation of high concentration of welding fume. Additionally, cement pneumoconiosis is a kind of diffuse pulmonary fibrosis caused by the long-term inhalation of cement dust, which belongs to silicate pneumoconiosis.

### 2.3. Statistical Analysis

Data sorting and descriptive analyses were performed using SPSS 20.0 (IBM, Armonk, NY, USA). 

## 3. Results

### 3.1. Changes in the Number of Pneumoconiosis Cases Reported from 2006 to 2017

**Figure 1:**
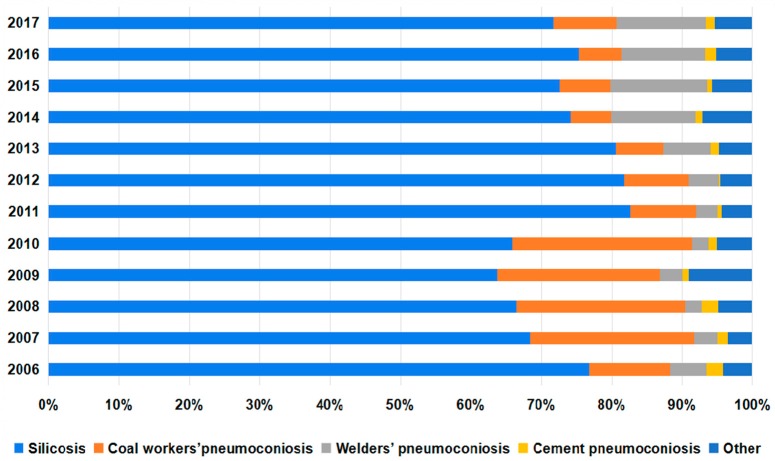
The changes in the number of pneumoconiosis cases reported from 2006 to 2017.

From 2006 to 2017, a total of 9243 cases of pneumoconiosis were reported in the Jiangsu Province. Silicosis (6732 cases, 72.8%) and CWP (1359 cases, 14.7%) accounted for 87.5% of the total reported pneumoconiosis. The main annually reported pneumoconiosis cases were silicosis and CWP. In recent years, the proportion of silicosis declined and that of welders’ pneumoconiosis cases gradually increased. Moreover, the number of cement pneumoconiosis cases slightly decreased (Figure 1 and Table S2).

The number of pneumoconiosis cases showed an overall decreasing trend. Since 2006, the number of silicosis cases increased at first, reaching the highest in 2010 (791 cases), then declining until it was stable at less than 500 cases in the last four years. CWP showed a similar trend as silicosis, the number of which increased until 2010 and then decreased gradually. In addition, there was a slowly rising trend in the number of cement pneumoconiosis, and the number of welders’ pneumoconiosis cases was relatively stable, with little fluctuation (Figure S1).

### 3.2. Changes in the Stages of Pneumoconiosis Cases Reported from 2006 to 2017

**Figure 2:**
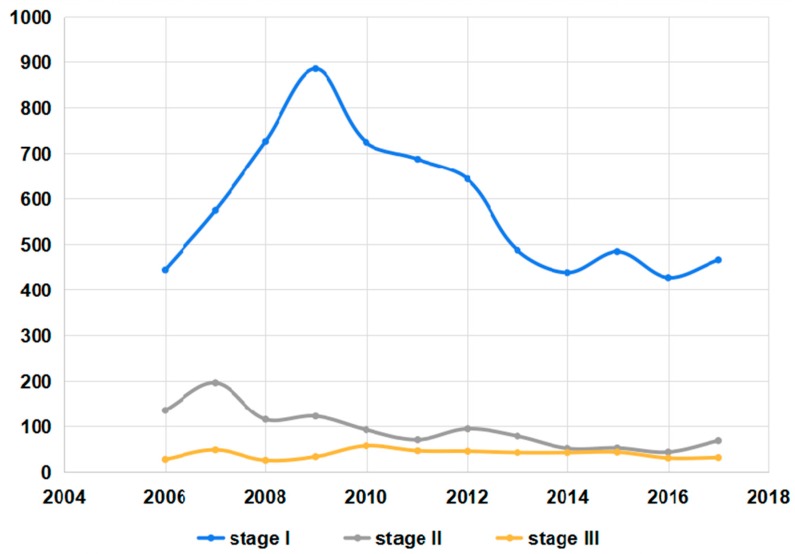
The changes in the stages of pneumoconiosis cases reported from 2006 to 2017.

Stage I pneumoconiosis patients accounted for approximately 75% of the total. The Pneumoconiosis cases in stage II gradually reduced. Meanwhile, the number of stage III patients showed a rising trend, but it decreased obviously in the last two years (Figure 2 and Table S3). Silicosis and CWP cases had a similar distribution and there was a gradual decline in the number of different stages. Welders’ pneumoconiosis cases were mainly in stage I and II. Cement pneumoconiosis patients were mostly in stage I (Table S4).

### 3.3. Regional Distribution of Pneumoconiosis Cases Reported from 2006 to 2017

**Figure 3:**
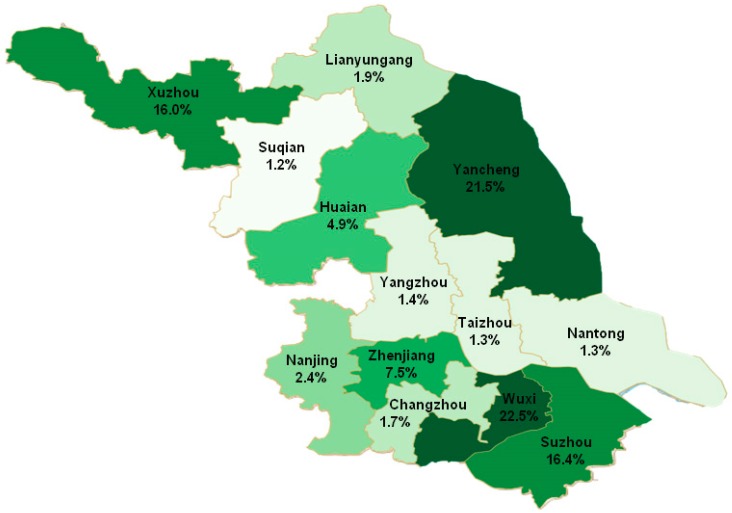
The regional distribution of pneumoconiosis cases reported from 2006 to 2017.

The top four cities reporting the most cases of pneumoconiosis were Wuxi (2076 cases, 22.5%), Yancheng (1990 cases, 21.5%), Suzhou (1512 cases, 16.4%) and Xuzhou (1482 cases, 16.0%). Taizhou, Nantong and Suqian, the three lowest ranking cities, had 124, 121 and 107 reported cases respectively (Figure 3). Totally 1783, 1461 and 1402 cases of silicosis patients were reported in Yancheng, Wuxi and Suzhou, accounting for 69% of silicosis cases. Additionally, 814 CWP cases (59.9%) were reported in Xuzhou. Welders’ pneumoconiosis cases were mainly reported in Wuxi (214 cases, 38.8%). Cement pneumoconiosis was mainly from Zhenjiang (54 cases, 47.8%) (Table 1).

### 3.4. Enterprise Ownership Type and Scale Distribution of Pneumoconiosis Cases Reported from 2006 to 2017

Between 2006 and 2017, most of the pneumoconiosis cases were reported in state-owned enterprises and collectively-owned enterprises, the number of which were 5396 (58.4%) and 2200 (23.8%) respectively. The pneumoconiosis cases in other forms of enterprise types accounted for about 16.8% (Table 2). In the terms of the enterprise scale, a larger proportion of pneumoconiosis cases were distributed in small enterprises (4164 cases, 45.1%) (Table 3).

### 3.5. Industry and Occupation Distribution of Pneumoconiosis Cases Reported from 2006 to 2017

**Figure 4:**
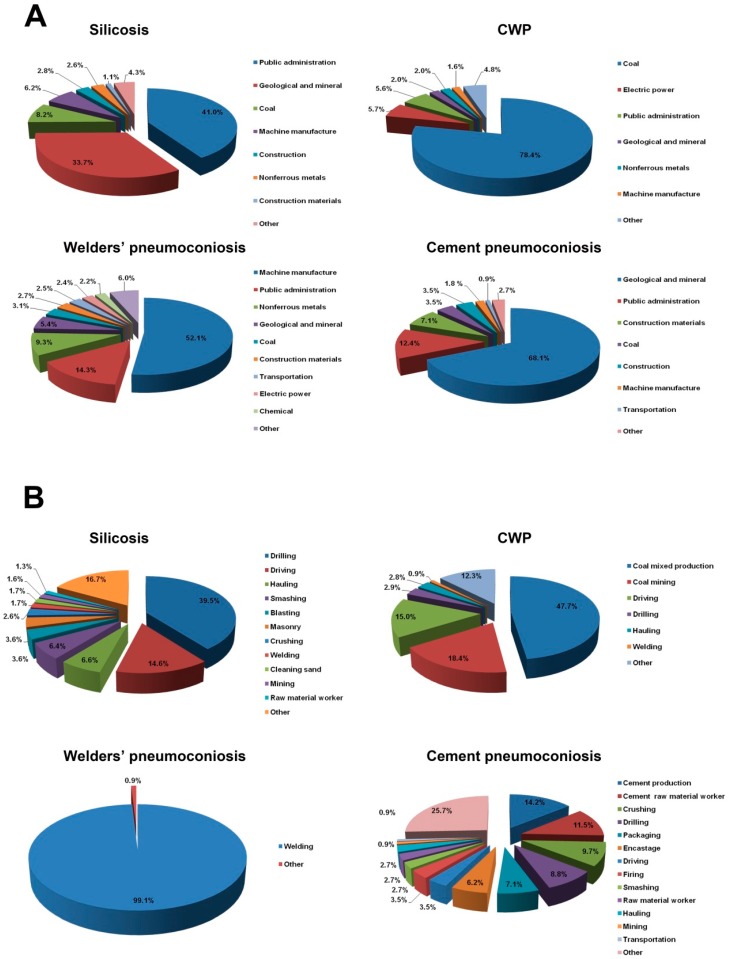
The industry and occupation distribution of pneumoconiosis cases reported from 2006 to 2017. (**A**) Industry distribution in different types of pneumoconiosis cases. (**B**) Occupation distribution in different types of pneumoconiosis cases.

All the pneumoconiosis cases were distributed in 24 industries. The cumulative cases mainly distributed in three industries: public administration (32.3%), the geological and mineral industry (27.6%) and the coal industry (17.8%). In the terms of silicosis, 41.0% of silicosis cases occurred in the public administration industry (2763 cases), followed by the geological and mineral industry which had 33.7% of total (2272 cases). In total, 1065 cases (78.4%) of CWP patients were from the coal industry. Welders’ pneumoconiosis patients were mainly from three industries, including the machine manufacture industry (52.1%), public administration (14.3%) and nonferrous metals (9.3%). Most of the cement pneumoconiosis cases (68.1%) were reported from the geological and mineral industry (Figure 4A, Tables S5–S8).

Further, pneumoconiosis occurred most frequently in the following occupations: drilling (30.3%), driving (9.3%), coal mixed production (7.2%), welding (6.1%) and smashing (5.2%). Silicosis cases were mostly distributed in drilling (2660 cases), driving (984 cases), hauling (447 cases) and smashing (430 cases), which accounted for 67.1% of the total CWP mainly that occurred in coal mixed work, coal mining and driving, the number of which were 648 (47.7%), 250 (18.4%) and 204 (15.0%), respectively. About 99.1% of welders’ pneumoconiosis patients were welders. Most of cement pneumoconiosis cases were engaged in cement related occupations (Figure 4B, Tables S9–S12).

### 3.6. The Overall Annual Trend of the Average Exposure Duration and Diagnosis Age of Pneumoconiosis Cases from 2006 to 2017

**Figure 5:**
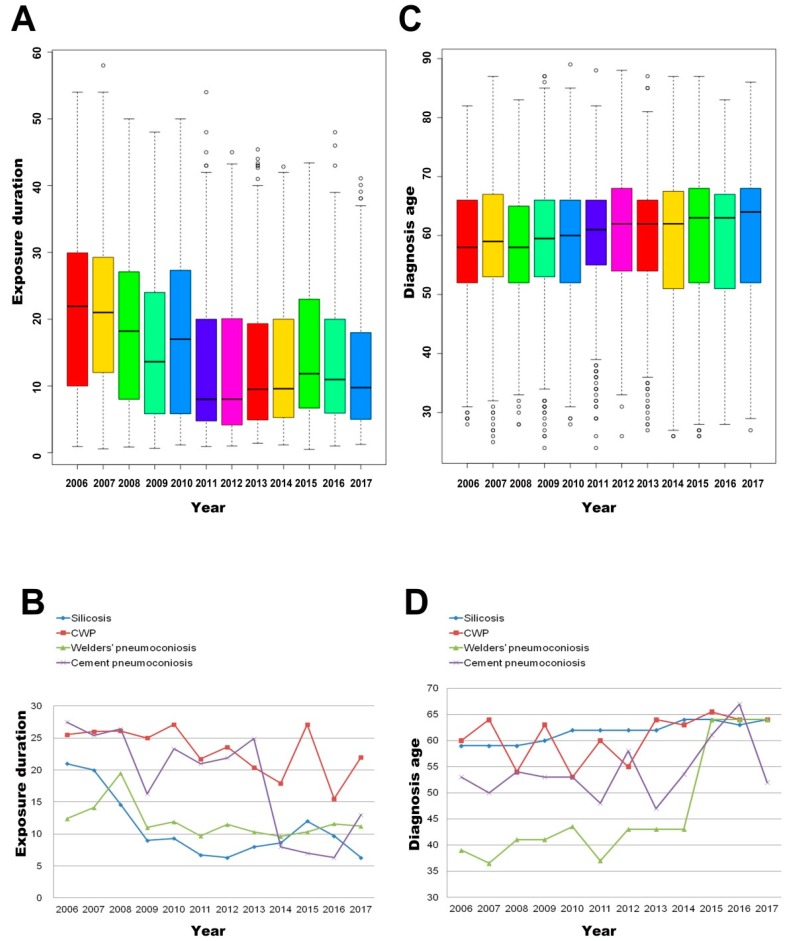
The overall annual trend of the average exposure duration and diagnosis age of pneumoconiosis cases from 2006 to 2017. (**A**) The overall annual trend of the average exposure duration of pneumoconiosis cases. (**B**) Changes in average exposure duration of different types of pneumoconiosis cases. (**C**) The overall annual trend of the average diagnosis age of pneumoconiosis cases. (**D**) Changes in the average diagnosis age of different types of pneumoconiosis cases.

The median exposure duration of the total pneumoconiosis cases was 13.2 years. The average exposure duration showed a downward trend, then rose slowly after 2012 (Table 4 and Figure 5A). For silicosis patients, the median exposure time, 10.2 years, was lowest among four types of pneumoconiosis. The largest median exposure duration was 25.3 years, which occurred in CWP cases; while for welders’ pneumoconiosis and cement pneumoconiosis, the medians of exposure duration were 11.0 years and 23.0 years, respectively (Table S13). In general, we observed a significantly shortened exposure time of the reported silicosis cases, which was 21.0 years in 2006 and reduced to its lowest value of 6.3 years in 2012. The exposure time of CWP and welders’ pneumoconiosis cases was slowly declining and then increased again. The cement pneumoconiosis exposure duration showed a downward trend generally (Figure 5B). 

The median age at diagnosis of reported pneumoconiosis cases was 61.0 years. The average diagnosis age increased slowly, and the median age of diagnosis increased from 58 years in 2006 to 64 years in 2017 (Table 5 and Figure 5C). The minimum median age at diagnosis of 45 years was found in welders’ pneumoconiosis cases, while the maximum median age at diagnosis of 62 years was found in patients with silicosis (Table S14). For silicosis cases, the diagnosis age showed a rising trend and its median increased from 59 years to 64 years. The age at diagnosis of CWP cases showed a volatile increase, being 60 in 2006 and reaching a minimum (53 years) in 2010 and then gradually increasing to about 65 years. Cement pneumoconiosis cases showed similar volatility in the increased trend with CWP; the diagnosis age of welders’ pneumoconiosis cases fluctuated around about 41 years old, but was up to over 60 in 2016 and 2017 (Figure 5D).

## 4. Discussion

Pneumoconiosis is one of the occupational diseases with the highest occurrence frequency in the world, especially in developing countries [16,17,18,19]. In order to understand the contributing factors and trends of pneumoconiosis as well as to provide new insight in developing more effective prevention and control measurements, we analyzed the reported pneumoconiosis cases in the Jiangsu Province from 2006 to 2017.

A total of 9243 pneumoconiosis cases were reported in the Jiangsu Province from 2006 to 2017, increasing at first and then declined. The Occupational Disease Prevention and Control Law was promulgated in 2002 in China. After the publicity and implementation of the law, the relevant policies were further implemented basically around 2005. Additionally, in order to strengthen the surveillance for occupational diseases, the Network Direct Report System of Occupational Diseases was constructed in 2006 in China. Thus, the increased reported pneumoconiosis patients may be the previous cumulative cases which were diagnosed after 2006. Moreover, the new Diagnostic Criteria of Pneumoconiosis (GBZ70-2009) has been implemented since 2009, which requires relatively more frequent health examinations for observation objects. As a result, many enterprises took the initiative to request the re-diagnosis of workers exposed to dust. Besides, the number of workers who take the initiative to ask for diagnosis gradually increased due to the increasing awareness of self-protection. These factors might result in an increase in pneumoconiosis cases around 2009.

It was found that the dust concentration in a mine of Jiangsu decreased significantly from 1975 to 2014, while the exceeded standard rates of dust concentration were still more than 40% [20]. Moreover, an analysis on the occupational exposure to dust and harmful gas in adults aged 40 years and older in China showed that the exposure rate of dust and/or harmful gas was 46.3%, while the exposure protection rate was low. Although lower than that in the western area, the exposure rate in the eastern areas was still more than 40% [21]. This evidence suggested that the health hazards caused by dust was still severe. 

Among the various pneumoconiosis cases, silicosis and CWP accounted for the majority, almost 87.5%. Based on our results, in the past twelve years, the silicosis cases in the Jiangsu Province accounted for 72.9% of the total pneumoconiosis cases, the ratio of which was significantly higher than that of the nation (39.7%) and other provinces like Hubei (28.9%), Hebei (62.3%), Anhui (47.7%), indicating that the situation in the Jiangsu Province is even more serious than other regions [15,22]. CWP was relatively smaller, accounting for only 14.7%. It may be associated with a smaller proportion of the coal mining industry in Jiangsu due to the relatively diversified industrial structure. Therefore, silicosis and CWP were the most popular pneumoconiosis diseases, indicating that coal and silica dust are currently the most dangerous occupational hazards in the Jiangsu Province. In addition, pneumoconiosis cases induced by welding and cement dust increased slightly in recent years, requiring more attention.

The regional distribution of pneumoconiosis cases was also closely related to the industry structure. More than 70% of pneumoconiosis cases were silicosis in Wuxi, Yancheng and Suzhou City. This is probably associated with the population size, the industry structure, the economic type and the ability to prevent and control occupational diseases in a region. In addition, there were a large number of sappers participating in national defense projects in Yancheng in later 1950s to 1970s. These sappers were not diagnosed with silicosis until they sought treatment for other complications after being insulated from dust exposure for several years, which was in agreement with what was reported in the relevant research literature [14]. Thus, there were higher rates of silicosis in Yancheng. In Xuzhou, over 50% of pneumoconiosis cases were CWP since the largest industry in Xuzhou was coal production. Additionally, Xuzhou’s CWP cases accounted for 59.9% of the total CWP cases in Jiangsu, which was consistent with the distribution of coal mines in the Jiangsu Province [23].

There are more pneumoconiosis cases in state-owned enterprises compared to other enterprises. For one thing, there is a relatively larger base of dust workers in state-owned enterprises. For another thing, state-owned enterprises do better in the prevention and control of occupational diseases, so pneumoconiosis patients have been detected, diagnosed and reported in time.

The cases of pneumoconiosis in Jiangsu are mainly concentrated in industrial systems and related occupations. Among them, silicosis cases were mainly found in the public administration industry (41.0%) and geological/mineral industry (33.7%), most of the cases in public administration were sappers from the local civil administration department; the CWP cases were mainly from coal industry (78.4%). According to the distribution of occupations, we found that silicosis cases were mainly distributed in drilling, driving, hauling and smashing and CWP cases were mainly engaged in coal mixed work, coal mining and driving. In addition, since reported pneumoconiosis cases were mainly from small enterprises, extensive attention is required to prevent and control pneumoconiosis in small enterprises.

Our results showed a decreasing trend of the exposure duration of reported pneumoconiosis and also the silicosis cases, which implies that the risk of dust is becoming more and more serious. Some factors are considered to account for this trend. First of all, a dose-response relationship between dust exposure level and the incidence of pneumoconiosis has been reported previously [24]. The increasing exposure level of the workers may result from the high level of dust concentration in the workplace, long work time and the lack of effective personal protective measures. It was reported that many tatami production workers were diagnosed as pneumoconiosis, their average age and exposure duration at diagnosis were shorter in Jiangsu. This is because most of the tatami enterprises were small (less than 50 employees), the workers need to work for 8–10 h every day without effective protection [25]. At the same time, the fact that dust concentrations in the workplace exceed the occupational exposure limits is still widely seen in China, and the standard of dust concentration in China is more lenient than that in other countries [17,26,27], which would inevitably lead to a large number of patients with pneumoconiosis. This reveals the necessity to study and adjust the standard of occupational exposure limits in China. Additionally, the functions of supervision and management were transferred from the Department of Health to Production Safety Supervision Department in 2010, surveillance of occupational diseases intensified. With the popularization of relevant laws and the strengthening of workers’ awareness of self-protection, the occupational health examination of dust workers is more frequent and timely than that in the past, which makes the exposure duration relatively short. What is more, the records of occupational history were not complete. Since the diversified forms of labor and employment, as well as the increasing mobility of workers, some pneumoconiosis patients had worked in more than one enterprise. However, some patients were unwilling to provide accurate occupational history and only provided information about their last job due to the issue of compensation for occupational diseases, which might lead to statistical bias on the onset age. In addition, the quality and accuracy of the occupational disease report system are still incomplete. In fact, it has been pointed out that more than 90% of cases have not been included in the existing occupational disease report database [28]. These results suggested that the existing occupational disease reporting system could not fulfill the role of reflecting the burden of occupational diseases in China.

Some suggestions are concluded based on the above results. First, the occupational health management of key industries and working types should be strengthened according to the characteristics and trends of pneumoconiosis. Second, it is fundamental to decrease dust concentration and workers’ exposure level in the workplace, among which engineering protection is the key part [29]. Third, it is of great importance to implement national occupational health standards strictly, as well as to explore the suitable prevention and control models for the Jiangsu Province. In addition, measures should be taken to strengthen the legal restraint to the enterprises. 

There are some limitations to this study. Our analysis is in accordance with the reporting time of the pneumoconiosis cases, but in general, there would be delays in reporting, about 85% to 95% of pneumoconiosis cases were reported in the year of diagnosis or the year after. What is more, although reflecting a certain trend, these data are still influenced by many factors, which need further analysis.

In summary, the morbidity of pneumoconiosis in Jiangsu was still severe. The pneumoconiosis cases were concentrated in several regions, industries, and work types. Moreover, the patients had a short diagnosis age length. In addition, the high incidence rate of silicosis, CWP, cement pneumoconiosis and welders’ pneumoconiosis need to be given more attention. This study provides insight into the current situation of pneumoconiosis and highlighted the need for effective pneumoconiosis control strategies in preventing pneumoconiosis and protecting workers’ health.

## Figures and Tables

**Table 1:** The regional distribution of pneumoconiosis cases reported from 2006 to 2017.

Silicosis		Coal Workers’ Pneumoconiosis		Welders’ Pneumoconiosis		Cement Pneumoconiosis		Other		Total	
n	%	n	%	n	%	n	%	n	%	n	%
1461	21.7	198	14.6	214	38.8	7	6.2	196	40.2	2076	22.5
1783	26.5	93	6.8	51	9.3	21	18.6	42	8.6	1990	21.5
1402	20.8	13	1.0	35	6.4	6	5.3	56	11.5	1512	16.4
604	9.0	814	59.9	39	7.1	8	7.1	17	3.5	1482	16.0
446	6.6	104	7.7	48	8.7	54	47.8	41	8.4	693	7.5
343	5.1	44	3.2	29	5.3	10	8.8	31	6.4	457	4.9
145	2.2	28	2.1	24	4.4	4	3.5	17	3.5	218	2.4
142	2.1	9	0.7	5	0.9	1	0.9	15	3.1	172	1.9
128	1.9	9	0.7	15	2.7	0	0.0	9	1.8	161	1.7
41	0.6	28	2.1	44	8.0	1	0.9	16	3.3	130	1.4
76	1.1	5	0.4	19	3.4	0	0.0	24	4.9	124	1.3
65	1.0	9	0.7	25	4.5	1	0.9	21	4.3	121	1.3
96	1.4	5	0.4	3	0.5	0	0.0	3	0.6	107	1.2

**Table 2:** The enterprise ownership type distribution of pneumoconiosis cases reported from 2006 to 2015.

Enterprise Ownership	Silicosis		Coal Workers’ Pneumoconiosis		Welders’ Pneumoconiosis		Cement Pneumoconiosis		Other		Total	
	n	%	n	%	n	%	n	%	n	%	n	%
State-owned	3757	55.8	1260	92.7	170	30.9	34	30.1	175	35.9	5396	58.4
Collective	2037	30.3	34	2.5	36	6.5	7	6.2	86	17.6	2200	23.8
Private	841	12.5	40	2.9	310	56.3	29	25.7	193	39.5	1413	15.3
Foreign	48	0.7	21	1.5	30	5.4	36	31.9	25	5.1	160	1.7
Hong Kong, Macao and Taiwan	19	0.3	0	0.0	3	0.5	2	1.8	2	0.4	26	0.3
Other types	30	0.4	4	0.3	2	0.4	5	4.4	7	1.4	48	0.5

**Table 3:** The enterprise scale distribution of pneumoconiosis cases reported from 2006 to 2015.

Enterprise Size	Silicosis		Coal Workers’ Pneumoconiosis		Welders’ Pneumoconiosis		Cement Pneumoconiosis		Other		Total	
	n	%	n	%	n	%	n	%	n	%	n	%
Large	749	11.1	896	65.9	64	11.6	7	6.2	43	8.8	1759	19.0
Medium	490	7.3	261	19.2	141	25.6	60	53.1	79	16.2	1031	11.2
Small	3577	53.1	98	7.2	269	48.8	23	20.4	197	40.4	4164	45.1
Micro	367	5.5	18	1.3	35	6.4	9	8.0	24	4.9	453	4.9
Unknown	1549	23.0	86	6.3	42	7.6	14	12.4	145	29.7	1836	19.9

**Table 4:** The changing trend of the average exposure duration of pneumoconiosis cases from 2006 to 2017.

Year	n	Mean (SD)	Range	Median	Quantile
2006	685	20.7 (11.6)	0.9–54.0	21.9	(10.0, 29.9)
2007	904	20.9 (11.0)	0.6–58.0	21.0	(12.0, 29.3)
2008	802	18.3 (10.8)	0.8–50.0	18.2	(8.0, 27.1)
2009	842	15.3 (10.3)	0.7–48.0	13.6	(5.8, 24.0)
2010	1201	17.1 (11.1)	1.2–50.0	17.0	(5.8, 27.3)
2011	841	12.8 (10.2)	0.9–54.0	8.0	(4.8, 20.0)
2012	874	12.7 (10.4)	1.0–45.0	8.0	(4.2, 20.1)
2013	670	12.9 (9.8)	1.4–45.4	9.5	(4.9, 19.3)
2014	603	13.3 (9.7)	1.2–42.8	9.6	(5.3, 20.0)
2015	665	15.1 (10.0)	0.5–43.4	11.8	(6.7, 23.0)
2016	500	13.5 (9.6)	1.0–48.0	11.0	(5.9, 20.0)
2017	656	12.4 (9.1)	1.3–41.1	9.8	(5.0, 18.0)
Total	9243	15.7 (10.8)	0.5–58.0	13.2	(0.5, 58.0)

SD: Standard deviation.

**Table 5:** The changing trend of the average diagnosis age of pneumoconiosis cases from 2006 to 2017.

Year	n	Mean (SD)	Range	Median	Quantile
2006	685	58.2 (10.7)	28–82	58	(52, 66)
2007	904	59.0 (10.7)	25–87	59	(53, 67)
2008	802	57.7 (10.1)	28-83	58	(52, 65)
2009	842	59.0 (10.5)	24–87	60	(53, 66)
2010	1201	58.8 (10.3)	28–89	60	(52, 66)
2011	841	60.1 (9.9)	24–88	61	(55, 66)
2012	874	60.7 (10.4)	26–88	62	(54, 68)
2013	670	59.9 (10.5)	27-87	62	(54, 66)
2014	603	59.5 (11.6)	26–87	62	(51, 67.5)
2015	665	60.4 (11.7)	26–87	63	(52, 68)
2016	500	60.4 (10.9)	28–83	63	(51, 67)
2017	656	60.7 (11.0)	27–86	64	(52, 68)
Total	9243	59.4 (10.7)	24–89	61	(53, 67)

SD: Standard deviation.

## Supplementary Materials

The following are available online at http://www.mdpi.com/1660-4601/16/3/437/s1, Figure S1: Changes in the number of pneumoconiosis cases reported from 2006 to 2017, Table S1: Division standard of enterprise scale, Table S2: Changes in the number of pneumoconiosis cases reported from 2006 to 2017, Table S3: Changes in the stages of total pneumoconiosis cases reported from 2006 to 2017, Table S4: Changes in the stages of different pneumoconiosis cases reported from 2006 to 2015, Table S5: Industry distribution of silicosis cases from 2006 to 2017, Table S6: Industry distribution of CWP cases from 2006 to 2017, Table S7: Industry distribution of Welders’ pneumoconiosis cases from 2006 to 2017, Table S8: Industry distribution of Cement pneumoconiosis cases from 2006 to 2017, Table S9: Distribution of working types of silicosis cases from 2006 to 2017, Table S10: Distribution of working types of CWP cases from 2006 to 2017, Table S11: Distribution of working types of Welders’ pneumoconiosis cases from 2006 to 2017, Table S12: Distribution of working types of Cement pneumoconiosis cases from 2006 to 2017, Table S13: The trend of the average exposure duration of different pneumoconiosis cases from 2006 to 2015, Table S14: The trend of the average diagnosis age of different pneumoconiosis cases from 2006 to 2015.
