# Supplementary material for: Characteristics and Trends of Pneumoconiosis in the Jiangsu Province, China, 2006–2017
Source: Int J Environ Res Public Health. 2019 Feb 2;16(3):437. doi: 10.3390/ijerph16030437 (PMC6388371; doi:10.3390/ijerph16030437)
Supplement: Supplementary file 1 [file ijerph-16-00437-s001.pdf]

Supplementary figure

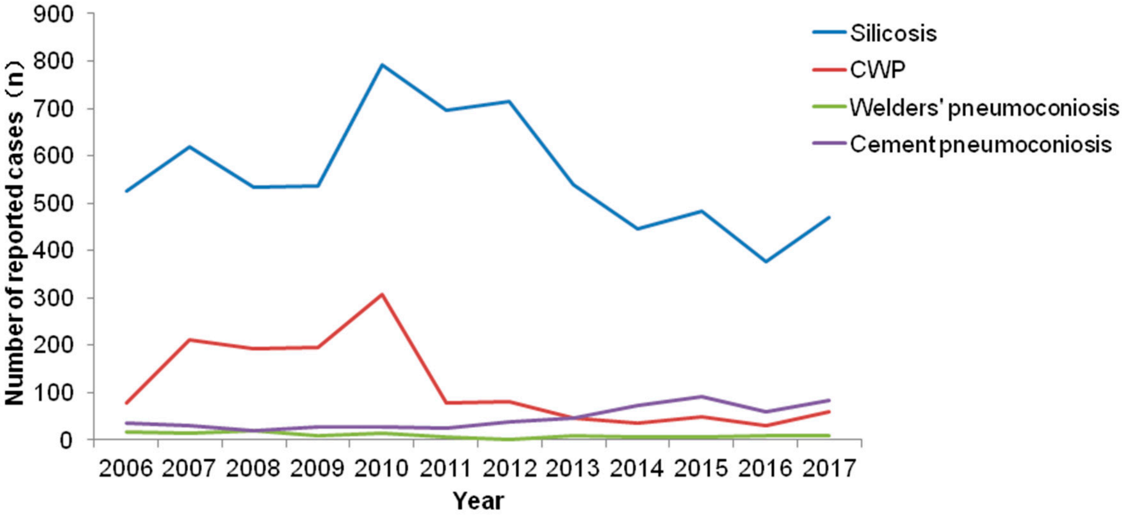

Figure S1. Changes in the number of pneumoconiosis cases reported from 2006 to 2017.

Changes in the classification of pneumoconiosis cases reported from 2006 to 2017

Supplementary Table

Table S1. Division standard of enterprise scale

Industry type	Large			Medium			Small			Micro		
	Operation revenue (million)	Employees (n)	Assets (million)	Operation revenue (million)	Employees (n)	Assets (million)	Operation revenue (million)	Employees (n)	Assets (million)	Operation revenue (million)	Employees (n)	Assets (million)
Agriculture, forestry, animal husbandry and fishery	⩾200	——	——	[5,200）	——	——	[0.5,5)	——	——	<0.5	——	——
Industry	⩾400	⩾1000	——	[20,400)	[300,1000)	——	[3,20)	[20,300)	——	<3	<20	——
Construction industry	⩾800	——	⩾800	[60,800)	——	[50,800)	[3,60)	——	[3,50)	<3	——	<3
Communications and transportation industry	⩾300	⩾1000	——	[30,300)	[300,1000)	——	[2,30)	[20,300)	——	<2	<20	——
Warehousing industry	⩾300	⩾200	——	[10,300)	[100,200)	——	[1,10)	[20,100)	——	<1	<20	——
Real-estate industry	⩾2000	——	⩾100	[10,2000)		[50,100)	[1,10)	——	[20,50)	<1	——	<20
Other	——	⩾300	——	——	[100,300)	——	——	[10,100)	——	——	<10	——

Table S2. Changes in the number of pneumoconiosis cases reported from 2006 to 2017

Year	Silicosis		CWP		Welders' pneumoconiosis		Cement pneumoconiosis		Other		Total	
	n	%	n	%	n	%	n	%	n	%	n	%
2006	526	76.8	79	11.5	35	5.1	16	2.3	29	4.2	685	100.0
2007	618	68.4	211	23.3	30	3.3	13	1.4	32	3.5	904	100.0
2008	533	66.5	192	23.9	19	2.4	19	2.4	39	4.9	802	100.0
2009	537	63.8	194	23.0	27	3.2	8	1.0	76	9.0	842	100.0
2010	791	65.9	307	25.6	28	2.3	14	1.2	61	5.1	1201	100.0
2011	695	82.6	79	9.4	25	3.0	5	0.6	37	4.4	841	100.0
2012	715	81.8	80	9.2	37	4.2	2	0.2	40	4.6	874	100.0
2013	540	80.6	45	6.7	45	6.7	8	1.2	32	4.8	670	100.0
2014	447	74.1	35	5.8	72	11.9	6	1.0	43	7.1	603	100.0
2015	483	72.6	48	7.2	91	13.7	5	0.8	38	5.7	665	100.0
2016	377	75.4	30	6.0	59	11.8	8	1.6	26	5.2	500	100.0
2017	470	71.6	59	9.0	83	12.7	9	1.4	35	5.3	656	100.0
Total	6732	72.9	1359	14.1	551	6.4	113	1.3	488	5.3	9243	100.0

Table S3. Changes in the stages of total pneumoconiosis cases reported from 2006 to 2017

Year	New reported cases(n)	Stage I		Stage II		Stage III	
		n	%	n	%	n	%
2006	685	462	67.4	172	25.1	51	7.4
2007	904	583	64.5	258	28.5	63	7.0
2008	802	629	78.4	138	17.2	35	4.4
2009	842	623	74.0	154	18.3	65	7.7
2010	1201	981	81.7	133	11.1	87	7.2
2011	841	632	75.1	102	12.1	107	12.7
2012	874	662	75.7	133	15.2	79	9.0
2013	670	498	74.3	97	14.5	75	11.2
2014	603	449	74.5	78	12.9	76	12.6
2015	665	502	75.5	86	12.9	77	11.6
2016	500	391	78.2	68	13.6	41	8.2
2017	656	507	77.3	96	14.6	53	8.1
Total	9243	6919	74.9	1515	16.4	809	8.8

Table S4. Changes in the stages of different pneumoconiosis cases reported from 2006 to 2015

Year	Silicosis						CWP						Welders' pneumoconiosis						Cement pneumoconiosis					
	Stage Ⅰ		Stage Ⅱ		StageⅢ		Stage Ⅰ		Stage Ⅱ		StageⅢ		Stage Ⅰ		Stage Ⅱ		StageⅢ		Stage Ⅰ		Stage Ⅱ		StageⅢ	
	n	%	n	%	n	%	n	%	n	%	n	%	n	%	n	%	n	%	n	%	n	%	n	%
2006	345	69.5	139	26.4	42	8.0	55	69.6	18	22.8	6	7.6	27	77.1	8	22.9	0	0.0	15	93.8	1	6.2	0	0.0
2007	383	65.5	179	29.0	56	9.1	136	64.5	70	33.2	5	2.4	25	83.3	5	16.7	0	0.0	13	100.0	0	0.0	0	0.0
2008	406	80.3	101	18.9	26	4.9	170	88.5	20	10.4	2	1.0	19	100.0	0	0.0	0	0.0	19	100.0	0	0.0	0	0.0
2009	390	80.7	99	18.4	48	8.9	146	75.3	35	18.0	13	6.7	25	92.6	2	7.4	0	0.0	7	87.5	0	0.0	1	12.5
2010	629	82.1	97	12.3	65	8.2	277	90.2	15	4.9	15	4.9	21	75.0	6	21.4	1	3.6	12	85.7	2	14.3	0	0.0
2011	522	75.1	85	12.2	88	12.7	57	72.2	6	7.6	16	20.3	18	72.0	6	24.0	1	4.0	5	100.0	0	0.0	0	0.0
2012	552	77.2	94	13.1	69	9.7	55	68.8	21	26.2	4	5.0	25	67.6	11	29.7	1	2.7	1	50.0	0	0.0	1	50.0
2013	401	74.3	76	14.1	63	11.7	29	64.4	9	20.0	7	15.6	41	91.1	4	8.9	0	0.0	8	100.0	0	0.0	0	0.0
2014	317	70.9	62	13.9	68	15.2	26	74.3	4	11.4	5	14.3	69	95.8	2	2.8	1	1.4	6	100.0	0	0.0	0	0.0
2015	342	70.8	74	15.3	67	13.9	34	70.8	6	12.5	8	16.7	91	100.0	0	0.0	0	0.0	5	100.0	0	0.0	0	0.0
2016	287	76.1	54	14.3	36	9.5	24	80.0	5	16.7	1	3.3	57	96.6	1	1.7	1	1.7	7	87.5	1	12.5	0	0.0
2017	357	76.0	67	14.3	46	9.8	46	78.0	10	16.9	3	5.1	79	95.2	4	4.8	0	0.0	4	44.4	4	44.4	1	11.1
Total	4931	73.2	1127	16.7	674	10.0	1055	77.6	219	16.1	85	6.3	497	90.2	49	8.9	5	0.9	102	90.3	8	7.1	3	2.7

Table S5. Industry distribution of silicosis cases from 2006 to 2017

Indusrty	Number of cases (n)	Percentage(%)
Public administration	2763	41.0
Geological and mineral	2272	33.7
Coal	550	8.2
Machine manufacture	420	6.2
Construction	189	2.8
Nonferrous metals	178	2.6
Construction materials	71	1.1
Other	289	4.3
Total	6732	100.0

Table S6. Industry distribution of CWP cases from 2006 to 2017

Indusrty	Number of cases (n)	Percentage(%)
Coal	1065	78.4
Electric power	77	5.7
Public administration	76	5.6
Geological and mineral	27	2.0
Nonferrous metals	27	2.0
Machine manufacture	22	1.6
Other	65	4.8
Total	1359	100.0

Table S7. Industry distribution of Welders' pneumoconiosis cases from 2006 to 2017

Indusrty	Number of cases (n)	Percentage(%)
Machine manufacture	287	52.1
Public administration	79	14.3
Nonferrous metals	51	9.3
Geological and mineral	30	5.4
Coal	17	3.1
Construction materials	15	2.7
Transportation	14	2.5
Electric power	13	2.4
Chemical	12	2.2
Other	33	6.0
Total	551	100.0

Table S8. Industry distribution of Cement pneumoconiosis cases from 2006 to 2017

Indusrty	Number of cases (n)	Percentage(%)
Geological and mineral	77	68.1
Public administration	14	12.4
Construction materials	8	7.1
Coal	4	3.5
Construction	4	3.5
Machine manufacture	2	1.8
Transportation	1	0.9
Other	3	2.7
Total	113	100.0

Table S9. Distribution of working types of silicosis cases from 2006 to 2017

Working type	Number of cases (n)	Percentage(%)
Drilling	2660	39.5
Driving	984	14.6
Hauling	447	6.6
Smashing	430	6.4
Blasting	245	3.6
Masonry	243	3.6
Crushing	174	2.6
Welding	114	1.7
Cleaning sand	114	1.7
Mining	107	1.6
Raw material worker	87	1.3
Other	1127	16.7
Total	6732	100.0

Table S10. Distribution of working types of CWP cases from 2006 to 2017

Working type	Number of cases (n)	Percentage(%)
Coal mixed production	648	47.7
Coal mining	250	18.4
Driving	204	15.0
Drilling	40	2.9
Hauling	38	2.8
Welding	12	0.9
Other	167	12.3
Total	1359	100.0

Table S11. Distribution of working types of Welders' pneumoconiosis cases from 2006 to 2017

Working type	Number of cases (n)	Percentage(%)
Welding	546	99.1
Other	5	0.9
Total	551	100.0

Table S12. Distribution of working types of Cement pneumoconiosis cases from 2006 to 2017

Working type	Number of cases (n)	Percentage(%)
Cement production	16	14.2
Cement raw material worker	13	11.5
Crushing	11	9.7
Drilling	10	8.8
Packaging	8	7.1
Encastage	7	6.2
Driving	4	3.5
Firing	4	3.5
Smashing	3	2.7
Raw material worker	3	2.7
Hauling	3	2.7
Mining	1	0.9
Transportation	1	0.9
Other	29	25.7
Total	113	100.0

Table S13. The trend of the average exposure duration of different pneumoconiosis cases from 2006 to 2015

Year	Silicosis				CWP				Welders' pneumoconiosis				Cement pneumoconiosis			
	n	Mean (SD)	Range	Median	n	Mean (SD)	Range	Median	n	Mean (SD)	Range	Median	n	Mean (SD)	Range	Median
2006	526	20.1 (12.2)	0.9-54.0	21.0	79	25.4 (8.5)	3.8-43.9	25.5	35	16.4 (9.3)	5.8-40.3	12.4	16	28.0 (5.8)	15.9-35.5	27.5
2007	618	20.1 (11.6)	0.6-58.0	20.0	211	24.4 (8.8)	2.2-42.5	26.0	30	16.8 (8.6)	6.1-35.0	14.1	13	23.8 (5.9)	7.7-30.0	25.4
2008	533	15.8 (10.9)	0.8-50.0	14.6	192	23.9 (8.7)	2.8-39.0	26.1	19	18.0 (7.9)	4.1-29.2	19.5	19	25.9 (5.7)	15.2-38.5	26.4
2009	537	12.7 (9.7)	0.7-48.0	9.0	194	23.9 (8.2)	3.5-43.4	25.0	27	12.9 (7.1)	3.6-33.3	11.0	8	18.2 (9.6)	5.1-29.5	16.3
2010	791	13.9 (10.9)	1.2-50.0	9.3	307	25.3 (7.6)	2.5-39.8	27.1	28	16.1 (10.5)	4.0-33.7	11.9	14	21.7 (6.9)	7.9-31.8	23.3
2011	695	11.6 (9.8)	0.9-54.0	6.7	79	21.6 (9.9)	4.0-39.8	21.7	25	11.4 (7.4)	2.2-35.0	9.7	5	20.2 (9.4)	6.0-30.0	21.0
2012	715	11.5 (10.1)	1.0-45.0	6.3	80	22.1 (10.0)	3.5-38.0	23.6	37	12.5 (6.2)	3.0-28.0	11.5	2	21.9 (7.5)	16.6-27.2	21.9
2013	540	12.2 (9.9)	1.4-45.4	8.0	45	20.4 (9.0)	2.8-36.5	20.4	45	13.0 (7.5)	2.8-42.7	10.3	8	22.1 (7.9)	4.0-29.2	24.9
2014	447	12.7 (9.9)	1.2-42.8	8.6	35	18.8 (9.0)	4.0-36.8	17.9	72	11.8 (6.4)	2.1-32.7	9.6	6	10.6 (7.0)	4.0-21.3	8.0
2015	483	15.0 (10.4)	0.5-43.4	12.0	48	26.1 (7.5)	6.9-38.3	27.1	91	11.3 (3.9)	6.8-27.1	10.3	5	10.5 (7.5)	3.2-20.0	7.0
2016	377	13.5 (10.2)	1.0-48.0	9.7	30	17.5 (8.7)	6.0-34.0	15.5	59	12.2 (4.7)	5.1-26.8	11.6	8	7.0 (2.2)	4.3-10.8	6.3
2017	470	11.4 (9.3)	1.5-41.1	6.3	59	20.9 (8.6)	3.0-36.0	22.0	83	12.2 (4.3)	4.3-26.2	11.2	9	12.9 (10.6)	1.3-33.1	13.0
Total	6732	14.2 (10.9)	0.5-58.0	10.2	1359	23.7 (8.7)	2.2-43.9	25.3	551	13.0 (6.7)	2.1-42.7	11.0	113	20.4 (9.3)	1.3-38.5	23.0

*SD: Standard deviation

Table S14. The trend of the average diagnosis age of different pneumoconiosis cases from 2006 to 2015

Year	Silicosis				CWP				Welders' pneumoconiosis				Cement pneumoconiosis			
	n	Mean (SD)	Range	Media n	n	Mean (SD)	Rang e	Media n	n	Mean (SD)	Rang e	Median	n	Mean (SD)	Range	Median
2006	526	59.3 (9.4)	29-82	59	79	60.5 (12.6)	32-81	60	35	40.8 (10.5)	28-76	39	16	52.8 (4.4)	43-60	53
2007	618	59.1 (9.5)	25-85	59	211	62.6 (10.6)	33-87	64	30	40.4 (9.9)	28-61	36.5	13	48.3 (6.3)	39-58	50
2008	533	59.0 (9.9)	28-83	59	192	55.7 (8.9)	31-81	54	19	42.4 (8.6)	28-57	41	19	53.6 (8.4)	43-73	54
2009	537	59.6 (8.3)	28-85	60	194	61.8 (11.1)	35-85	63	27	39.7 (10.5)	24-67	41	8	57.0 (13.1)	46-86	53
2010	791	61.3 (8.8)	29-89	62	307	54.7 (10.8)	35-85	53	28	42.3 (12.0)	28-71	43.5	14	54.6 (9.2)	43-82	53
2011	695	61.5 (8.3)	29-82	62	79	59.5 (12.4)	36-88	60	25	38.2 (9.2)	24-66	37	5	52.4 (12.3)	39-69	48
2012	715	62.2 (9.2)	33-88	62	80	58.9 (11.0)	40-88	55	37	42.3 (6.2)	26-63	43	2	58.0 (5.7)	54-62	58
2013	540	61.7 (9.3)	27-87	62	45	61.6 (10.6)	38-79	64	45	42.5 (7.8)	28-64	43	8	49.6 (6.3)	45-64	47
2014	447	62.7 (9.2)	27-87	64	35	61.5 (11.4)	42-82	63	72	41.2 (6.9)	26-63	43	6	55.5 (8.3)	46-70	53.5
2015	483	63.5 (9.3)	26-87	64	48	64.8 (10.5)	40-82	65.5	91	42.4 (6.4)	26-61	64	5	63.6 (5.2)	59-72	61
2016	377	59.7 (10.9)	28-83	63	30	61.8 (9.2)	43-78	64	59	60.2 (11.7)	32-78	64	8	67.0 (5.8)	60-75	67
2017	470	50.5 (11.0)	27-86	64	59	61.5 (11.7)	29-86	64	83	62.5 (10.3)	38-81	64	9	58.0 (11.7)	45-74	52
Tota l	673 2	60.8 (9.5)	25-89	62	135 9	59.1 (11.3)	29-88	58	551	42.8 (12.6)	24-81	45	113	54.8 (9.3)	39-86	53

*SD: Standard deviation
